# Correction of a Reference

**DOI:** 10.1001/jamanetworkopen.2019.1971

**Published:** 2019-03-22

In the Invited Commentary titled “The Rise of Wearable Technology in Health Care,”^1^ published February 1, 2019, the elocator and doi numbers in the first reference were incorrect. The full reference should have appeared as follows: “Daskivich TJ, Houman J, Lopez M, et al. Association of wearable activity monitors with assessment of daily ambulation and length of stay among patients undergoing major surgery. *JAMA Netw Open*. 2019;2(2):e187673. doi:10.1001/jamanetworkopen.2018.7673.” This article has been corrected.^1^
